# The Effects of *Astragalus membranaceus* Active Extracts on Autophagy-Related Diseases

**DOI:** 10.3390/ijms20081904

**Published:** 2019-04-17

**Authors:** Hao Shan, Xueping Zheng, Min Li

**Affiliations:** School of Pharmaceutical Sciences, Sun Yat-Sen University, Guangdong Provincial Key Laboratory of New Drug Design and Evaluation, Guangzhou, Guangdong 510006, China; 17827063336@163.com (H.S.); jnzxp1993@126.com (X.Z.)

**Keywords:** autophagy, *Astragalus membranaceus*, *Astragalus membranaceus* extracts, autophagy dysregulation-associated diseases

## Abstract

Autophagy is an evolutionarily conserved ‘self-eating’ process that maintains cellular, tissue, and organismal homeostasis. New studies on autophagy, mediated by subsets of autophagy proteins, are emerging in many physiological and pathological processes. *Astragalus* *membranaceus* (AM), also named *Huangqi*, is one of the fundamental herbs in traditional Chinese medicine and its extracts have been proved to possess many biological activities related to autophagy, including anti-oxidation, anti-inflammation, anticancer, anti-photoaging, and improvement of cardiomyocyte function. Evidence suggests that AM extracts can have therapeutic potential in autophagy dysregulation-associated diseases because of their biological positive effects. Here we will review the literature concerning the effects of AM extracts on autophagy dysregulation-associated diseases.

## 1. Introduction

Autophagy is a process mediated by subsets of evolutionarily conserved genes from yeast to higher eukaryotes [1]. Autophagy responds to various environmental cues and maintains cellular, tissue, and whole-body homeostasis through regenerating metabolic precursors and clearing subcellular debris [2]. *Astragalus membranaceus* (AM), as a fundamentally representative herb of traditional Chinese medicine, contains numerous components and has a wide range of biological activities [3,4,5]. In addition to the expanding roles of autophagy in human health and diseases, recent developments have begun to reveal the close interaction between autophagy and AM-mediated biological activities in human diseases. Here we aim to address the expanding mechanisms involved in the interaction between the two fields.

## 2. Autophagic Pathways and Human Diseases

**Figure 1:**
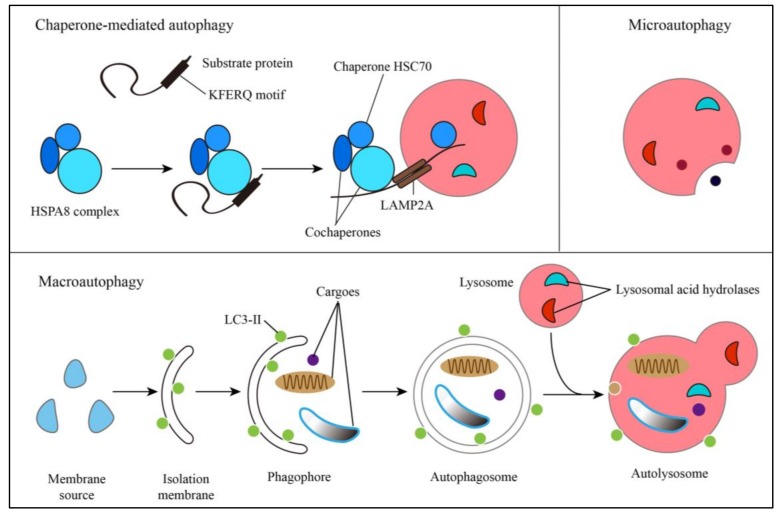
Three types of autophagy. In the major autophagic pathway, various organelles, including the endoplasmic reticulum (ER), mitochondria, mitochondria-associated membranes (MAMs), the Golgi, the plasma membrane and recycling endosomes could supply membrane sources for nucleation of the isolation membranes (originally termed the phagophore), which eventually develop into autophagosome through elongation. Mature autophagosome fuses with a lysosome to form an autolysosome, in which the cargoes sequestered by the autophagosome is degraded and released into the cytoplasm for use in cellular processes, including protein biosynthesis and energy production.

Three distinct types of autophagy have been found to help maintain cellular homeostasis, including chaperone-mediated autophagy, microautophagy, and macroautophagy (Figure 1). Chaperone-mediated autophagy selectively degrades KFERQ-like motif-bearing proteins, which are delivered to the lysosomes via the HSPA8 complex (chaperone HSC70 and cochaperones) [6]. The concept of microautophagy is still not integrated and can be temporarily categorized into three types according to its form of membrane dynamics: microautophagy with lysosomal protrusion, lysosomal invagination, and endosomal invagination [7]. Macroautophagy is characterized by a double-membrane vesicle called an autophagosome, which unselectively or selectively sequesters cytosolic cargo. Autophagosomes deliver cargo to the lysosomes either directly or after fusion with endosomal compartments, and finally form the single-membrane autolysosomes. Contents of the autolysosomes are degraded by lysosomal acid hydrolases and released for metabolic recycling [8]. Among them, macroautophagy (hereafter referred to as autophagy) is the major pathway to renovate damaged proteins and organelles, respond to stress, and participate in physiological and pathologic processes [2].

Autophagy involves a set of conserved ATG genes, and the ATG proteins encoded by them function at different steps of autophagic pathways including initiation, nucleation, elongation, maturation, and degradation. Seven parts, constituting the whole autophagic pathway, include the ULK1 complex (involving ULK1, FIP200, ATG13, and ATG101), the PI3KC3-C1 complex (involving Beclin 1, VPS34,VPS15, and ATG14), the PI3KC3-C2 (involving Beclin 1, VPS34, VPS15, and UVRAG), ATG9A, WIPI1/2-ATG2A/B complex, the ubiquitin-like ATG12 conjugation system (involving ATG5, ATG7, ATG10, ATG12, and ATG16L1), and the ubiquitin-like LC3 conjugation system (involving ATG3-ATG12, ATG5-ATG12-ATG16L1, ATG3, and ATG4B) [1].

Autophagy plays crucial roles in human health and participates in a wide range of human diseases, including cancer, neurodegenerative diseases and aging, pulmonary diseases, metabolic diseases, vascular diseases, and infectious diseases. Readers interested in autophagy-related diseases can refer to recent reviews [2,9,10].

## 3. AM and AM Extracts

AM is one of the most widely used fundamental herbs in traditional Chinese medicine and has a long history, showing numerous biological activities including immunomodulation, anti-inflammation, antioxidation, tonic action, hepatoprotection, diuresis, antidiabetes, anticancer, anti-photoaging, and expectorant action (Table 1). More than 200 compounds have been isolated and identified from AM. The major active extracts include triterpene saponins, flavonoids, and polysaccharides (for reviews see [3,11,12]).

Triterpene saponins are major constituents of AM ranging from 0.5 mg/g to 3.5 mg/g [13]. Five major saponins (astragalosides I, II, and IV, and isoastragaloside I and II) represent more than 80% of the total *Astragalus* triterpene saponins, and astragaloside IV (AS-IV) is known as the qualitative control biomarker [14].

More than 63 flavonoids are contained in AM, within the range of 0.5‒3.0 mg/g [15]. Among them, isoflavones are the major constituents and calycosin-7-O-β-D-glucoside is known as the qualitative control biomarker [13,16] (Pharmacopoeia Commission of the People’s Republic of China, 2015).

Polysaccharides contained in AM are macromolecules with complicated chemical structures, such that it is more difficult to isolate and identify their individual constituents. Fourteen polysaccharides have been identified and 13 of them have β-D-(1→3)-galactan moieties branched with β-D-(1→3)-galactooligosaccharide side-chains [17]. Knowledge of *Astragalus* polysaccharides (APS) and qualitative control methods is still poor.

Other components found in AM include amino acids, fatty acids, and trace elements. Some of them were isolated and identified in the past few years. Although some trace components such as bifendatatum [18] and lipoxygenase [19] have been demonstrated to mediate specific functions, the biological activities of many other components in AM are still unclear.

## 4. Expanding Mechanisms of Autophagy in AM Extracts

**Figure 2:**
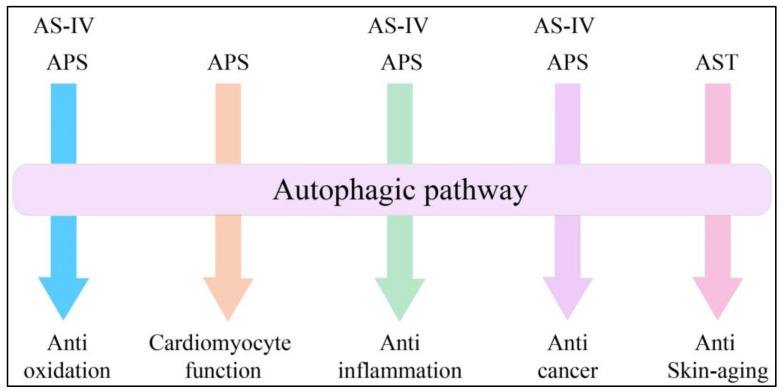
The pathological conditions where autophagy was shown to be involved in AM effects. AM extracts show expanding effects on autophagy dysregulation-associated diseases. AS-IV and APS both have effects on oxidation, inflammation and cancer through regulation of autophagy. APS could also play a protective role in DOX-induced cardiotoxicity, while AST exerts antagonistic effect on UVB-induced photoaging.

Ever since the basic machinery of autophagy was demonstrated to be conserved and widely implicated in human diseases [29], our understanding of AM active constituents on autophagy-related diseases has been dramatically expanding (Figure 2). Moreover, the mechanisms of AM extracts affecting autophagy have begun to be revealed.

### 4.1. Advances in Anti-Oxidation

AS-IV has been reported to exert anti-oxidation activity in various types of cells, partly owing to its protective effects against mitochondrial dysfunction [30,31,32,33]. The mitochondria, as a target and the major cellular source of reactive oxygen species (ROS), can be cleared by mitophagy while undergoing mitochondrial damage. Mitophagy selectively sequesters dysfunctional or superfluous mitochondria, thus fine-tuning mitochondrial number and preserving energy metabolism. Although the mechanisms by which autophagosomes identify and selectively target damaged mitochondria are still unknown, the major steps of the mitophagic pathway are similar to those for macroautophagy [34,35].

A study found that, in rat vascular smooth muscle cells, AS-IV significantly increased mitochondrial oxygen consumption rates, ATP production, the mitochondrial DNA levels, thus reversing the angiotensin II-induced mitochondrial morphological changes, the increase in NADPH oxidase and xanthine oxidase activity, the production of ROS, the decrease in mitochondrial membrane potential, and manganese superoxide dismutase activity [36]. During this beneficial AS-IV-mediated process, the protein levels of peroxisome receptor-gamma coactivator-1α, mitochondrial transcription factor A, parkin, and dynamin 1-like protein 1 (Drp1) in rat vascular smooth muscle cells increased [36]. Among these affected proteins, parkin and Drp1 are vital to mitophagy [34,37,38]. In addition, a previous study has demonstrated that increased ROS levels in the mitochondrial matrix resulted in mitochondrial damage and the subsequent activation of parkin-dependent mitophagy [39]. In conclusion, it is possible that AS-IV exerts anti-oxidation activity against mitochondrial dysfunction and excessive ROS generation through regulating mitophagy.

APS was reported to inhibit the autophagy induced by peroxide injury in C2C12 myoblasts through the mTOR-independent pathway [40]. Oxidative stress, structural muscle damage, and muscle inflammation can produce excessive ROS including hydrogen peroxide (H_2_O_2_), superoxide (O_2_^−^), hydroxyl radical (·OH) and singlet oxygen (1/2 O_2_) [41,42], which has been demonstrated to be involved partly in the development of skeletal muscle atrophy both in vitro and in vivo [43,44]. In models of C2C12 myoblasts treated with H_2_O_2_, APS significantly inhibited autophagy, which was characterized by a reduced number of autophagosomes under electron microscopy and the ratio of LC3-II/LC3-I in a Western blotting analysis. However, the expression of p-p70S6K and p70S6K remained unchanged in C2C12 myoblasts after treatment with APS, which means this inhibitory process is independent of mTOR [40]. Soon after this discovery, researchers focused on another ROS-producing process, and found that in an excessive exercise model of mice based on chronic fatigue, expression of the autophagy markers Atg7 and LC3 was strongly induced, whereas the expression of p62 was decreased. Mitochondrial dysfunction and the morphological changes induced by oxidative stress during this process could be ameliorated by APS. Autophagy during this process was also inhibited and was independent of AMPK, a vital regulator of the energy response and activator of mTOR [45]. They also found that, in a chronic kidney disease-related muscle wasting model, which was closely associated with oxidative stress [46], APS could improve muscle wasting through Akt/mTOR, ubiquitin proteasome and autophagy signaling, and SLC38A2 might be involved in disease progression due to the inhibitory effect of siRNA [47]. However, two fundamental aspects of this process remain unanswered: what is the actual contribution of oxidative stress to a series of reactions like skeletal muscle atrophy, tissue damage, and muscle dysfunction? What is the signaling pathways of APS-mediated anti-autophagy effect?

Moreover, a recent study found that selenizing APS could inhibit autophagy activated by H_2_O_2_ via PI3K/Akt activation, thus attenuating the porcine circovirus type 2 replication promotion caused by oxidative stress [48]. Some other viruses could also utilize autophagy to facilitate their replication, such as enterovirus 71 [49] and classical swine fever virus [50]. However, whether these viruses’ replication could be attenuated by selenizing APS through regulation of autophagy remains unclear.

As mentioned previously, increased ROS levels can induce mitophagy [39]. However, the actual extents of mitophagy and nonselective autophagy during those ROS-producing processes are still unknown, and the expression levels of mitophagy-related proteins need further detection. Taken together, AS-IV and APS appear to exert anti-oxidation activity via regulating autophagy in different pathways, while undergoing ROS-induced oxidative stress.

### 4.2. Advances in Cardiomyocyte Function

Doxorubicin (DOX) is one of the most effective anticancer drugs [51]. However, DOX’s dose-dependent cardiotoxicity, including severe cardiomyopathy and congestive heart failure, restricts its clinical application [52]. A recent study demonstrated that DOX could induce heart failure by disturbing cardiomyocyte autophagic flux, which led to excessive accumulation of autophagosomes and autophagic cell death, while APS could restore impaired cardiomyocyte autophagic flux to normal level and improve cardiomyocyte function through the regulation of the AMPK/mTOR pathway [53]. Despite significant progress in discovering the relationship between APS and autophagy in cardiomyocyte function, two fundamental aspects of this process remain unanswered: what is the exact role of autophagy in DOX-induced cardiotoxicity and APS-mediated protective effect? Can the autophagy involved in this process be regarded as mitophagy? It is now well established that impaired mitochondria could be cleared by mitophagy and defects in mitophagy result in mitochondrial dysfunction [34,54]. Taken together, considering the critical role of mitochondria in the heart, it is very likely that DOX impairs mitophagic activity, which creates an imbalance in mitochondrial dynamics and defects in mitochondrial respiration, and finally leads to cardiotoxicity. On the contrary, APS might reverse this process through regulation of mitophagy, thus improving cardiomyocyte function.

### 4.3. Advances in Anti-Inflammation

AS-IV was recently found to exert anti-inflammation activity through disrupting the crosstalk between autophagy and the pancreatic ER stress kinase-eukaryotic initiation factor 2 alpha (PERK-eIF2α) pathway while undergoing heat-induced thermal inhalation injury [55], which is the main cause of pulmonary diseases [56]. In a heat exposure damage model based on 16HBE140 cells, ER stress was observed, which was characterized by the increased expression of ER stress molecular chaperones GPR78 and ER stress-associated apoptosis protein CHOP. Treatment with AS-IV significantly reduced autophagosome puncta and the expression of GPR78, CHOP, phosphorylated PERK, phosphorylated eIF2α, Beclin 1 and ratio of LC3-II/LC3-I. Another surprising finding is that the ER stress inhibitors GSK2656157 and eIF2α siRNA reversed the excessive autophagy induced by heat exposure. In addition, ER stress inhibitors and autophagy inhibitors used in this model all suppressed the release of inflammatory factors including IL-6, IL-8, IL-10, TNF-α, and TGF-β [55]. Taken together, AS-IV could disrupt the crosstalk between autophagy and ER stress to be protective in inflammatory diseases.

In another interesting study based on a CCl_4_-induced hepatocellular necrosis model, APS highly decreased the mRNA expression levels of ATG7 and the ratio of LC3-II/LC3-I. Despite this kind of extract showing strong protective effects against CCl4-induced hepatocellular necrosis, along with numerous inflammatory cytokines and chemokines, future work to understand the exact role of autophagy during this process would expand on and explain these observations [57].

Sometimes, an increase in autophagy also contributes to the reduction of inflammation. A recent study observed a significant increase of Beclin 1 and LC3-II along with a remarkable decrease of p62 in both IL-1β-stimulated rheumatoid arthritis fibroblast-like synoviocytes and RSC-364 cells after APS treatment, suggesting APS induces autophagy in rheumatoid arthritis. In IL-1β-stimulated rheumatoid arthritis fibroblast-like synoviocytes, inhibition of autophagy by 3-methyladenine partly reversed APS-induced decrease of cell viability, increase of cell apoptosis, and repression of proinflammatory cytokines production. Moreover, in RSC-364 cells, the expression levels of p-AKT and p-mTOR decreased in a dose-dependent manner [58]. These results suggested that APS could suppress cell growth and the inflammatory response, partly by enhancing autophagy involving the PI3K/Akt/mTOR pathway.

### 4.4. Advances in Anticancer Treatment

A recent study based on rats with N-methyl-N’-nitro-N-nitrosoguanidine-induced gastric precancerous lesions revealed that AS-IV significantly decreased the ratio of LC3-II/LC3-I, and the expression levels of Ambra1, Beclin 1, p62, ATG5 and ATG12 proteins, compared with the model group. In conclusion, AS-IV could protect from gastric mucosal injury in this model, partly through regulation of autophagy. Gastric cancer has evolutionary histopathological stages, starting with chronic gastritis, followed by gastric precancerous lesions, including chronic atrophic gastritis, intestinal metaplasia, dysplasia, even carcinoma [59]. However, the role of AS-IV-mediated autophagy and its exact mechanism in gastric precancerous lesions and gastric cancer are still under exploration.

Besides the extracts’ own potential to treat cancer, APS might also enhance the antitumor effects of some existing drugs such as apatinib and cisplatin, which may involve the autophagic pathway. Recent studies found that apatinib caused significant inhibition of cell proliferation, migration, and invasion in pancreatic cells, which could be enhanced by APS through the further downregulation of p-AKT, p-ERK, and MMP-9. Although both apatinib and APS induced cellular autophagy in a dose-dependent manner, apatinib combined with APS did not significantly elevate the expression of LC3-II compared with the apatinib-treated group in ASPC-1, one of the human pancreatic cancer cell lines, but elevated it in PANC-1 [60]. They also showed that APS could enhance the antitumor effects of apatinib, partly via inhibition of the AKT signaling pathway in gastric cancer AGS cells, and cell proliferation was more significantly inhibited while suppressing autophagy by 3-methyladenine [61].

Another interesting study revealed that APS might increase the sensitivity of cervical cancer HeLa cells to cisplatin by enhancing HeLa cells’ autophagic activity. Its mechanism was probably correlated with the upregulation of Beclin 1, along with the downstream changes of proteins including the increased ratio of LC3-II/ LC3-I, and the downregulation of p62 [62].

Due to the complexity of diverse cancers, we speculated that the extents of anticancer effects of autophagy are obviously distinct among cancer cells. APS might be able to enhance chemotherapeutic agents’ anticancer function. However, sometimes autophagy might play dual roles and APS-mediated regulation of autophagy to certain levels could inhibit cell proliferation. It may have a signaling pathway that could regulate APS-mediated autophagy in cancer. Although such observations cannot be explained now, evidence from previous research shows that APS plays a role in the anticancer process [60,61,62].

### 4.5. Advances in Skin Aging

The skin is the largest organ of the human body and protects us from a wide range of potential external stressors like ultraviolet radiation and mechanical, chemical, and biological insults. Furthermore, the skin acts as one of our most important sense organs and its role in maintaining tissue and whole-body homeostasis is more critical than usual when undergoing aging [63]. Skin aging can be divided into two types according to the origin: intrinsic skin aging and extrinsic skin aging.

Intrinsic skin aging is an inevitable gene-related biological process, which is mediated and even controlled by the length and integrity of telomeres [64,65]. It is characterized by unblemished, smooth, pale, dry, less elastic skin with fine wrinkles. In spite of the unclear interactions with other aging organs, intrinsic aging usually occurs within the skin itself, via reductions in dermal mast cells, fibroblasts, collagen production, and flattening of the dermal‒epidermal junction/loss of rete ridges [66,67]. A recent article reported that autophagy is capable of restricting replicative crises, especially telomeric DNA damage while undergoing oncogenic transformation, by triggering autophagic cell death [68].

Extrinsic skin aging is the cumulative effect of extrinsic factors. Environmental factors, lifestyle-related factors, systemic morbidities, drugs, and other indirect factors all contribute differently to extrinsic skin aging [63,69]. Among those expanding various factors, exposure to sunlight is still the most significant factor leading to extrinsic skin aging. Therefore, extrinsic skin aging is often referred to as photoaging [70]. Sunlight at the surface of the Earth consists of about 52‒55% infrared light, 44% visible light, and 3% ultraviolet (UV) light. Among them, the most important factor is chronic exposure of the skin to solar UV light. Owing to the complete absorption of UVC (200‒290 nm) by the ozone layer and atmosphere, UVR reaching the surface of the Earth consists of >95% UVA (320‒400 nm) and ~5% UVB (290‒320 nm) [63,71]. UVB affects both the epidermis and dermis, while UVA only affects the dermis to a significant extent [72].

Recently, an interesting study demonstrated that astragaloside (AST) could antagonize UVB-induced photoaging of rat dermal fibroblasts through enhanced autophagy. Results in this study suggested that UVB irradiation could inhibit autophagy, thus impairing collagen-I formation and exacerbating photoaging, while AST increased the expression and accumulation of collagen-I, and antagonized UVB-induced photoaging not only through ERK and p38 inhibition but also autophagy activation [73]. Taken together, the present study provides the first evidence that AST protected rat dermal fibroblasts from UVB-induced photoaging through the regulation of autophagy. 

## 5. Application of AM Extracts through Regulation of Autophagy: A Nexus for Protection or Therapeutics?

Autophagy was originally studied in yeast and mammalian cells as a stress-response pathway. During the past few decades, interest in defining its roles in human health and diseases has spread. Currently, therapeutic targeting of autophagy in human diseases is limited by an incomplete understanding of the mechanisms involved in physiological and pathological conditions, a lack of compounds that can specifically regulate autophagy, and the limited availability of candidate therapeutics with clinical efficacy. Some compounds targeting different autophagy-related proteins have been reported to have surprising biological activities and thus represent useful prospects, such as Spautin (a Beclin 1 inhibitor) [74] and S130 (an ATG4B inhibitor) [75]. Also, some extracts of traditional Chinese medicine like AM have the potential to supply candidates for this purpose. So, it is essential to investigate the mechanisms of AM extract-mediated autophagy in diverse diseases.

Recently, some discoveries have shown a fascinating area of future research based on the interaction between autophagy and AM-mediated biological activities.

SH003, a formula of extracts isolated from AM, *Angelica gigas*, and Trichosanthes Kirilowii Maximowicz with a 1:1:1 ratio (w/w) has been revealed to inhibit tumor growth and invasion on highly metastatic breast cancer cells by inducing autophagy, both in vivo and in vitro without toxicity [76]. Although SH003 has been demonstrated to induce autophagy by inhibiting activation of STAT3- and mTOR-mediated signaling pathways, there is still a long way to explain the underlying mechanism and be applied for breast cancer.

AS-IV-mediated activation of autophagy was found likely to promote skin flap survival, which may relate to its anti-oxidation activity [77]. Whether its protective role in tissue damage via regulation of autophagy can be applied in clinical therapy remains unclear.

AST also performs its anti-photoaging effects and accelerates col1 formation through the regulation of autophagy [73]. Based on these findings, AST could have good potential as an anti-photoaging agent for therapy purpose. Accordingly, further studies using in vivo animal models need to be considered.

If the advances in these two aspects continue at an accelerated pace, agents coming from AM extracts that act on autophagy may eventually provide useful protection and therapeutics.

## 6. Concluding Remarks

Our summary of current work on the interaction between the autophagy process and the cellular pathways activated by AM extracts emphasizes the complexity of the effects of AM extracts on autophagy dysregulation-associated diseases. The studies on the relationship between AM and autophagy have only begun in recent years and the evidence is insufficient for us to make an integrated conclusion. What is becoming clear is the exquisite specificity that governs autophagic responses from the signals involved in the machinery mediating the formation of autophagosomes and how these responses assume essential roles in many aspects of the cell, and even the whole-body physiology. Although AM extracts have been demonstrated to exert effects on autophagy dysregulation-associated diseases, many questions remain to be answered about the exact molecular mechanisms of autophagy and AM-mediated pathways. Future work will provide more details, which would make important contributions to the understanding of the exact significance of the effects.

## Figures and Tables

**Table 1:** Representative extracts of AM.

Categories	Bioactive Chemical Constituents
Triterpene saponins [13,14,20,21]	Astragalosides Acetylastragaloside Isoastragaloside Astramembrannin Cycloastragenol Cyclosieversigenis Soyasaponin Soyasapogenol Lupeol
Flavonoids [13,15,16,22,23]	Isoflavonones Isoflavans Pterocarpans Flavonones Chalcones
Polysaccharides [17,24,25,26]	Glucans Heteropolysaccharide
Other components [18,27,28]	Phytosterols Volatile oil Fatty acids Amino acids Trace elements
